# Practice of Medicine and Surgery in Modern Times; Sins and Delinquencies of Professional Men

**Published:** 1862-12

**Authors:** 


ARTICLE XLIII.

### PRACTICE OF MEDICINE AND SURGERY IN MODERN TIMES; SINS AND DELINQUENCIES OF PROFESSIONAL MEN.

Practicing medicine and surgery in these modern days, is not calculated to make the physician pleasant and agreeable under all circumstances. The excitement incident to the condition of the government at this time;—the general deterioration as regards the moral honesty of mankind in the various departments of life;—the frequent solicitations on the part of that portion of community whose duty it is to replenish the earth, for medicine or means to sacrifice the delicate germ of embryotic life, (a crime of great magnitude, and rapidly on the increase: confined to no one class of society; the aristocratic, those who pine in want and penury, those within the pale of modern Christianity, as well as those who never felt the invigorating influence of a true religion; all, all are alike guilty of the heinous and unwarranted sin against the beautiful and benificent laws of nature, and of nature's God.) The many cares and anxious thoughts attending the ardous duties of the profession —the misery one is compelled to witness in the daily rounds— the want of appreciation of services manifested by many, and upon too many occasions; are all calculated to blunt the finer sensibilities of human nature, and change the character to such a degree that soon man is lost in the mere automaton. The profession is filled to overflowing; and envy and jealousies arise among practitioners, which feeling is fostered and cherished to such an extent that the people wonder why the profession tolerates it, and enquire if there is any honesty or nobleness of soul within the pale of the medical profession. So many and varied are the obstacles met with in the life and duties of the physician, therefore, that I conclude that he who can live in accordance with the ten commandments while pursuing his vocation, is made of sterner Christian material than the average of mankind. If such there be in the profession, they are entitled to a free-ticket to the realms of the future world, and may well and truly, "Thank God that they are not as other men are."

With the best of intentions as regards the profession and mankind in general, and with the idea (perhaps a vain one,) that the medical practitioner may be benefitted morally and professionally by a faithful and truthful portrayal of the many obstacles met with in practice, combined with the exposition of the sins and medical delinquencies of some medical men in every locality. I have ventured out in a new and untried field of labor.

As your numerous readers may enquire who I am, and desire to know upon what basis I stand and essay to the world an article on the *morale* of the profession; I shall only say, that I consider myself an old practitioner, although my head is not yet silvered with the rapid exit of "many years." And I console myself with the idea that I have, by reading and observation, acquired some knowledge of medical science, as well as become a proficient in judging of human character as found in sickness and health. I may also, perhaps, be characterized as a "medical heretic," as I belong to no pathy or ism, and never have, although a graduate of a respectable medical college. Consider me then, kind reader, an outsider, gathering knowledge from all available sources, and storing the mind with all useful material (gleaned from books and observation,) for the successful treatment of disease in all its varied forms. It has been my province to observe closely the effects of nature, to admire her successful efforts in restoring the human system to healthy action, when invaded by disease, and to select remedies to aid, rather than to direct or go before her. And he who sits quietly by, carefully noting her restorative powers in disease, or where accident or the surgeon's knife has produced serious and extensive wound of muscle and bone, and does not conclude that his skill is nothing in comparison to that of the *vis medicotri natusœ*, must be possessed of an organ of self-esteem that would set at defiance the will of the Almighty, or attempt to thwart the wicked designs of the devil.

But many there are, and of every pathy and ism, (mere pretenders, however,) who boastfully proclaim upon all occasions wonderful cures, for the purpose of gaining notoriety, and to increase their business. One will notice that this class of men will have any number of patients with heart disease, or phthisis pulmonalis, who recover in a few weeks under their treatment, and then go about proclaiming the surprising skill of the doctor. I have in my mind's eye numerous cases that have been diagnosed as organic diseases of the heart, which have been removed by attention to the digestive organs and moderate exercise, after being under the care of these deceitful practitioners for many months. Others make surgery their hobby, and though ignoring the first principle of the science, and devoid morally of every element that goes to make the man and true surgeon, are ever ready to sacrifice the patient to gain the eclat of the public, as operators. So great is this desire in the brain of many, that I have known preparations made and the operations commenced for the removal of what had been diagnosed as fatty tumors of the thigh and other parts, when the history of the case, and every appearance of the patient indicated the correct character of the disease. A few strokes of the knife, making deep and extensive wounds, discovers to the vision of the operators large abscesses. But with this class of men, such glaring demonstration of ignorance does not deter them from still boasting of their surgical skill; still continuing to deceive the public, and subjecting their followers to pain and suffering—all for name and notoriety. Can such men be true physicians and surgeons? Should they be tolerated by the medical profession, or their sins and medical delinquencies be passed over in silence? Alas, it is too often the case, but the welfare and progress of medical science and surgery forbid it. The welfare of the public, whose guardian the true physician is, demands this exposure, and it is a shameful neglect of duty not to do it.

To illustrate more pointedly the course of such professional men, I will suppose a case, which, if it should meet the eye of some, may bring to their minds patients sacrificed by their own practice, so similar in every respect as to lead them to conclude that it was "an o'er true tale." Having been requested to assist in the removal of a tumor on the upper part of the thigh of a young lady, I consented. On visiting the patient I found her one of those frail objects of humanity whom the Almighty had visited upon, to an alarming extent, the curse of scrofula.

The pale physiognomy, the blue veins coursing over the pallid marble-like forehead, the flabby muscular tissue, the sharp, full blue eye, the long eyelashes, the whitened blue skin, with several extensive cicatrices about the neck and joints, told but too plainly that death had secured another victim; it being only a matter of time when she would be called to that higher, and we trust a better world. Upon examining the tumor, its elastic feel; the colorless surface and the fluctuation, gave unmistakable evidence of the true character of it. My opinion not being solicited, it was not given, (having long since learned not to volunteer advice;) and the patient was prepared for the operation; chloroform administered, and my position assigned. The operator now enquired what kind of an incision he should make. I simply replied, "that an eliptical one would be proper for a fatty tumor of such a size in that location." The incisions were accordingly made six or eight inches in length, the dissection commenced, when, for the first time, the idea seemed to flash across the vision of the operator that the *fatty tumor had degenerated into an abscess*, and he incontinently exclaimed, "I do not know what we have got here." That was evident thought I, and loudly replied, "you will find out when the scalpel penetrates the abscess;" which it did the very next stroke. The pus poured suddenly forth in large quantities, and the astonished operator became nervous, and suggested that the wound be closed. I suggested that as the operation had proceeded so far, it would be well to evacuate the sac and dissect as much of it as could safely be done. My advice was followed, and the wound dressed. In explaining to the family he remarked, "that it was the first time he ever saw a fatty tumor degenerate into a scrofulous abscess." I replied, "that this one had done so very suddenly, as but a few minutes had elapsed since he had made his diagnosis." No further explanation was attempted, and I retired satisfied that the boastful operator's science and skill had signally failed in this case, as in many other instances perhaps, and he welcome to all the credit to be obtained under such circumstances. The readers may conclude that the imagination has largely supplied some of the immaterial portions of the above case; but let them look back over the practice of long years, and if they do not call to mind similar cases occurring in their own locality, they may deem themselves fortunate in having for their associates men of sterling worth, and free from the sins that are generated in the hearts and heads of too many professional men. But, nevertheless, I assure each and every one that there is more truth than poetry in the story.

I have mused over the strange conduct of this class of professional men, watched their erratic professional course through life, marked by ignorance and deceit, and contrasted it with the quiet unassuming manner of the really scientific and true physician and surgeon, and wondered why there should be such a difference in mankind. Doubting at the same time whether professional men of this character had human souls, or ever thought of reaching a future world, prepared to enjoy the happiness and glory allotted to the true physician, who is governed by principle in the social and professional circle, and acts in accordance with the requirements of the established code of medical ethics. M.

